# Psychometric properties of the brief fear of negative evaluation scale—Straightforward items

**DOI:** 10.3389/fpsyg.2025.1572752

**Published:** 2025-06-13

**Authors:** Greta Baldanzini, Gioia Bottesi, R. Nicholas Carleton, Umberto Granziol

**Affiliations:** ^1^Department of General Psychology, University of Padova, Padua, Italy; ^2^Department of Psychology, University of Regina, Regina, SK, Canada

**Keywords:** fear of negative evaluation (FNE), social anxiety disorder, social cognition, measurement invariance, BFNE-S

## Abstract

**Objectives:**

The present study evaluated the psychometric properties of the Brief Fear of Negative Evaluation Scale—straightforward items (BFNE-S) within an Italian sample. Specifically, the study was designed to validate the scale factor structure, reliability, concurrent validity, and measurement invariance across sexes.

**Method:**

A total of 652 participants (70.71% female and 29.29% males, aged 18–66) completed the BFNE-S and additional related scales including the Social Interaction Anxiety Scale (SIAS), Social Phobia Scale (SPS), Rosenberg Self-Esteem Scale (RSES), Depression Anxiety Stress Scales (DASS-21), and the SCOFF Questionnaire. Confirmatory factor analyses (CFA) were conducted to assess for a unidimensional structure, followed by testing of measurement invariance test between sexes. Reliability was evaluated using Cronbach's alpha and McDonald's Omega, and concurrent validity was tested through correlations with related measures.

**Results:**

The CFA supported the unidimensional structure of the BFNE-S with excellent fit indices (CFI = 0.998, RMSEA = 0.058). No measurement invariance violations were observed between sexes, despite their frequencies being slightly unbalanced. The BFNE-S demonstrated excellent internal consistency (Cronbach's α = 0.96, McDonald's ω = 0.97). There were positive correlations among BFNE-S, the SIAS (ρ = 0.67), SPS (ρ = 0.67), as well as with DASS-21 subscales (i.e., depression, anxiety, stress) and SCOFF, and inverse correlations with RSES.

**Conclusion:**

The BFNE-S exhibited robust reliability, validity, and measurement invariance across sexes in an Italian sample. Psychometric evidence supports the BFNE-S as a reliable tool for measuring fear of negative evaluation in the nonclinical population, providing a valuable resource for research and clinical assessments.

## Introduction

Fear of Negative Evaluation (FNE) is characterized by an intense and irrational fear of being scrutinized by others, along with distress over the possibility of negative judgment. FNE can be represented as a continuum, ranging from the absence of fear of negative judgment to extreme consequences (e.g., avoiding anything that may involve judgment) based on the different implicit threat values (i.e., the level of risk of being appraised by others) assigned to the stimuli (Watson and Friend, 1969; Weeks et al., 2009; Zhang et al., 2023).

FNE has consistently been associated with lower self-esteem, loneliness, social isolation, and increased sensitivity to rejection (Leary and Kowalski, 1995; Kocovski and Endler, 2000; Cheng et al., 2015), suggesting an association of FNE with psychopathologies such as eating disorders, depression, and particularly social anxiety (Utschig et al., 2010; Levinson and Rodebaugh, 2012, 2016; Mancuso et al., 2022; Preston et al., 2023). Indeed, FNE is a hallmark criterion of social anxiety disorder (SAD) (Wells et al., 1995; Rapee and Heimberg, 1997). SAD involves a pronounced and persistent fear of being judged—either positively (FPE) or negatively (FNE)—as well as concerns about interacting with strangers in social settings (Weeks et al., 2008a,b; American Psychiatric Association, 2013). The symptom profile can cause the avoidance of various social situations and substantially negatively impact quality of life, as patients frequently encounter interpersonal difficulties, struggle to maintain close relationships, and report reduced occupational or educational function (American Psychiatric Association, 2013; Alvi et al., 2022).

SAD has been a particularly prevalent mental disorder, with evidence of increases since the COVID-19 pandemic (Wells et al., 1995; Bandelow and Michaelis, 2015; Kindred and Bates, 2023). Positive and beneficial social interactions can be crucial for wellbeing (Segrin, 2001; Alden and Taylor, 2004; Chin et al., 2023; Monninger et al., 2023), which makes understanding the cognitive aspects of social anxiety (e.g., FNE) paramount for effective proactive and responsive intervention solutions to help mitigate SAD.

A commonly used instrument for measuring FNE is the Brief Fear of Negative Evaluation Scale (BFNE; Weeks et al., 2005). The BFNE is a short version of the 30-item scale designed by Watson and Friend (1969) that incorporates a wider range of response options using a 5-point Likert scale (from 0 “not at all characteristic of me” to 5 “entirely characteristic of me”). The BFNE is highly correlated with the original scale (*r* = 0.96; Leary, 1983), evidences moderate to high internal consistency across both clinical and nonclinical populations worldwide (α > 0.85; Weeks et al., 2005; Gallego Pitarch et al., 2007), high test-retest reliability (*r* = 0.75; Leary, 1983; Miller, 1995), and concurrent validity with measures of social anxiety (Wei et al., 2015). BFNE also demonstrates sensitivity to therapeutic changes among patients with SAD who receive cognitive behavioral therapy (Collins et al., 2005; Weeks et al., 2005). The reverse-worded items within the BFNE have consistently impacted the overall scores (Taylor, 1993; Carleton et al., 2011), leading to the development of three variations: the BFNE-R (Collins et al., 2005; Carleton et al., 2006), the BFNE-II (Carleton et al., 2007) and the BFNE-S (Rodebaugh et al., 2004; Weeks et al., 2005; Carleton et al., 2011).

The BFNE-R retained the original factor structure after revising the reverse-worded items to be straightforward (Collins et al., 2005; Carleton et al., 2006). The scale was eventually recommended against due to the instability in the factor structure introduced by the revised items (Carleton et al., 2011). The BFNE-II consists of seven straightforward items from the original BFNE and one revised item, and the BFNE-S includes only the eight straightforward items, which have been reported as more reliable and valid indicators of FNE (Rodebaugh et al., 2004; Weeks et al., 2005; Carleton et al., 2007). Compared to BFNE-II, the BFNE-S has demonstrated excellent internal consistency (α > 0.90), as well as strong factorial and construct validity in both undergraduate and clinical samples (Rodebaugh et al., 2004; Weeks et al., 2005; Norton and Weeks, 2009; Carleton et al., 2011). BFNE-S has also shown the highest validity for distinguishing SAD from other psychopathologies (Perczel-Forintos and Kresznerits, 2017; Fox et al., 2018).

The psychometric generalizability of the BFNE-S beyond North American samples remains unknown. Assessing whether BFNE-S robustly reflects a continuum between nonclinical and clinical populations across different countries is critical for understanding generalizability, facilitating international research and development efforts for proactive interventions and treatments, and supporting cross-cultural and multilingual meta-analytic results (Norton and Weeks, 2009; Pitarch, 2010; Boateng et al., 2018). There is also evidence of sex differences in social anxiety and social cognitions, with results suggesting female-specific correlations between anxiety and thoughts that may warrant targeted and customized interventions (Alvi et al., 2022). Sex differences align with previous research suggesting greater social cognitive abilities in women (Babchuk et al., 1985; Thayer and Johnsen, 2000). Consequently, investigating whether BFNE-S assesses FNE consistently across countries and sexes can provide novel pathways to address internationally underinvestigated elements of SAD (Harpole et al., 2015).

BFNE-S psychometric properties have been evaluated in Spain, Hungary, Turkey, and China (Pitarch, 2010; Perczel-Forintos and Kresznerits, 2017; Gur Kabul et al., 2023; Wei et al., 2015), but there are no studies yet with Italian data. Previous studies have used various samples, including nonclinical undergraduate and middle school students (Pitarch, 2010; Wei et al., 2015), as well as patients with anxiety disorders, mood disorders, and systemic sclerosis (Perczel-Forintos and Kresznerits, 2017; Gur Kabul et al., 2023). The unidimensional factor structure of the BFNE-S has thus far been supported, along with evidence of moderate positive correlations and therein concurrent validity among measures of social phobia (SP), major depressive disorder, and SAD, such as the Beck Depression Inventory (BDI), the Social Anxiety and Distress Scale (SAD), and the Rosenberg Self-Esteem Scale (RSES). To date, none of the international studies have assessed measurement invariance between sexes. The present study is designed to fill a gap in existing research by translating the BFNE-S into Italian, and assessing normative psychometric properties including reliability, concurrent validity, and measurement invariance using a nonclinical sample from Italy.

## Materials and methods

### Measures

*Brief Fear of Negative Evaluation Scale—straightforward items* (BFNE-S; Carleton et al., 2011): This scale assesses the degree of anxiety or apprehension experienced by an individual when anticipating negative evaluation from others. The BFNE-S includes the eight straightforward items (items 1, 3, 5, 6, 8, 9, 11, 12) from the original BFNE scale developed by Leary (1983). Responses are recorded on a five-point Likert scale, ranging from 0 (“not at all characteristic of me”) to 4 (“extremely characteristic of me”). BFNE-S has consistently showcased excellent internal consistency (α > 0.90), factorial and construct validity across undergraduate and clinical populations (Rodebaugh et al., 2004; Weeks et al., 2005; Norton and Weeks, 2009; Carleton et al., 2011). The translation process used in this study was guided by standard steps outlined in the psychology literature (Brislin, 1986). In the first step, three independent researchers translated the questionnaire from English to Italian and then agreed on a common version. The translation was done using idiomatic Italian appropriate for a sixth grade reading level. Furthermore, the researchers reviewed the common version to ensure that it did not contain colloquialisms, slang, or esoteric phrases that could complicate interpretation. The shared version was then back-translated by a bilingual individual with extensive knowledge of psychological research. The back-translation was found to be nearly identical to the original version.

*Social Interaction Anxiety Scale* (SIAS; Mattick and Clarke, 1998; Italian version: Sica et al., 2007): The SIAS is a self-report instrument that focuses on the level of fear triggered by social interactions, such as meeting new people at social gatherings. The 19 items are scored on a five-point Likert scale, ranging from 0 (“not at all characteristic or true of me”) to 4 (“extremely characteristic or true of me”). SIAS has shown high internal consistency (α = 0.93 and α = 0.89 in undergraduate and clinical samples, respectively), robust test-retest reliability, and validity (Orsillo, 2002; Rodebaugh et al., 2006). SIAS is commonly paired with the SPS to assess the symptoms of SA and SAD. Together, these tools have shown discriminant validity, effectively distinguishing patients with SAD from healthy controls and those with other anxiety disorders (Brown et al., 1997; Mattick and Clarke, 1998). Additionally, both scales are sensitive to symptom changes following cognitive-behavioral therapy for SAD (Mattick and Clarke, 1998). Within the Italian population, SIAS has shown high internal consistency (α = 0.86), test-retest reliability (*r* = 0.93), as well as moderate construct validity (Sica et al., 2007).

*Social Phobia Scale* (SPS; Mattick and Clarke, 1998; Italian version: Sica et al., 2007): The SPS assesses fear of being scrutinized or judged by others in everyday contexts, such as eating in public. The SPS is composed of 20 self-report items, rated on a Likert scale from 0 (“not at all characteristic or true of me”) to 4 (“extremely characteristic or true of me”). The SPS has demonstrated high internal consistency (α = 0.93 in undergraduate and clinical samples, α = 0.87 in the Italian sample), along with strong test-retest reliability (*r* = 0.87 in the Italian study) and validity (Orsillo, 2002; Sica et al., 2007). The Social Phobia Scale (SPS) and the Social Interaction Anxiety Scale (SIAS) were selected because fear of negative evaluation (FNE) is a core component of social anxiety, and both scales tap into different aspects of this domain: performance-related anxiety (SPS) and anxiety in interpersonal contexts (SIAS). These complementary perspectives allow for a more nuanced assessment of the convergent validity of the BFNE-S (Heimberg et al., 1992; Rodebaugh et al., 2004).

*Rosenberg Self-Esteem Scale* (RSES; Rosenberg, 1989; Italian version: Prezza et al., 1997). The RSES is a 10-item scale that assesses the global self-esteem of an individual. It includes five straightforward items to reflect positive self-esteem and five reversed items to indicate negative self-esteem. The responses range from “strongly disagree” (1) to “*strongly agree”* (4). Widely accepted as a measure of a unitary construct (Tomas and Oliver, 1999), the RSES has shown high internal consistency (α = 0.88) and good test-retest reliability (*r* = 0.82) even in an Italian sample (Fleming and Courtney, 1984; Rosenberg, 1989; Blascovich and Tomaka, 1993; Prezza et al., 1997; Schmitt and Allik, 2005).

*Depression Anxiety and Stress Scales-21 item version* (DASS-21; Lovibond and Lovibond, 1995; Italian version: Bottesi et al., 2015): The DASS-21 is a self-report questionnaire composed of three subscales that quantify symptoms of depression, anxiety, and stress. Each subscale contains seven items measured on a four-point Likert scale, from 0 (“did not apply to me at all”) to 3 (“applied to me very much, or most of the time”). The Italian version of the DASS-21 showed strong to excellent internal consistency, with Cronbach's alpha values ranging from α = 0.83 to α = 0.92 in clinical samples and from α = 0.74 to α = 0.90 in community samples. Additionally, it showed good convergent and divergent validity (Antony et al., 1998; Bottesi et al., 2015). DASS-21 was included to examine broader emotional correlates of FNE, as previous studies have shown that people with high FNE often exhibit elevated symptoms of depression, anxiety, and stress (Weeks et al., 2008a). These associations support the construct validity of the BFNE-S by situating FNE within the broader context of internalizing symptoms.

*The SCOFF Questionnaire* (Morgan et al., 1999; Italian version: Pannocchia et al., 2011): The SCOFF Questionnaire is a screening tool with five items that are designed to identify people at risk for anorexia and bulimia. Each item is rated with either 1 for “yes” or 0 for “no,” and total scores range from 0 to 5. A score of 2 or higher suggests an elevated risk of eating disorders. Although the SCOFF has demonstrated low reliability (α = 0.64), it has also shown high concurrent validity, good sensitivity, and specificity among individuals diagnosed with eating disorders (Hill et al., 2010; Pannocchia et al., 2011). The SCOFF questionnaire was used to explore potential associations between FNE and disordered eating behaviors. Research has suggested that social evaluative concerns can contribute to body dissatisfaction and maladaptive eating attitudes, especially among adolescents and young adults (Levinson and Rodebaugh, 2012, 2016). Including the SCOFF allows to investigate these broader implications of FNE beyond traditional social anxiety measures.

### Participants and procedure

Participants were recruited using a snowball sampling method by using social media (for example, Facebook and Instagram). After asking them to fill out an informed consent form, they were asked to provide sociodemographic information and to complete the present study questionnaires. Participants were asked to provide an email account to be re-contacted for the test-retest analyses. Participants were not paid for their participation. Exclusion criteria: age < 18 years, any kind of mental disorder ascertained by an expert, and above 30% of missing data on the entire battery of questionnaires. The study was approved by the Ethical Board of the University where one of the authors works, and it was conducted according to the Declaration of Helsinki.

### Data analysis

A confirmatory factor analysis (CFA) on the factor structure proposed by Carleton et al. (2011) was performed, followed by testing of measurement invariance in sex, reliability and validity analyses. The sample size estimation was conducted by setting a value of α = 0.05 and a power (i.e., 1–β) of 0.80.

#### Confirmatory factor analysis and measurement invariance

BFNE-S items were evaluated on a five-point Likert scale. Such a measure can be considered as an ordinal one. In fact, preliminary checks on the scale distributions suggested skewness and nonnormality. In similar scenarios, CFA models should be conducted employing proper estimators: in the present study, a weighted least squares with robust standard errors and a mean or mean and variance adjusted test statistic was selected (i.e., WLSMV). This choice aligns with previous recommendations (Schermelleh-Engel et al., 2003). Goodness-of-fit was assessed using the Comparative Fit Index (CFI) and the Root Mean Square Error of Approximation (RMSEA). Measurement invariance (MI) was tested to understand whether sex influenced participants' responses. Configural, metric, and scalar invariances were tested. Two criteria were used to compare the fits and determining the possible violations in measurement invariance: (a) the difference (Δ) between the fit indices, with CFI >0.01 and RMSEA >0.015, indicating violations of invariance, and (b) the overall fit of each model. Regarding the latter criterion, to define a fitting CFA model, whether the previous indices were above the respective thresholds (i.e., values < 0.9 for CFI and >0.1 for RMSEA; Hu and Bentler, 1999; Steiger, 1990), modification indices were examined to detect critical parameters. Partial measurement invariance was examined when an item caused misspecification in both the model tested on specific subsamples and in comparing MI models by relaxing only the parameters related to the critical item.

#### Reliability

Reliability was assessed using Cronbach's alpha and McDonald's Omega. A value ≥0.70 indicated adequate reliability. Test-retest reliability was also estimated on a subsample of 128 participants who filled the questionnaire after 1 month (Spearman' ρ).

#### Concurrent, convergent validity and linear models

Spearman**'s** ρ correlation coefficients were used to estimate validity between BFNE-S total score, SIAS, SPS (i.e., concurrent validity), RSES, SCOFF, and DASS-21 subscales for Anxiety, Depression, and Stress (i.e., convergent validity), with *p*-values adjusted using Bonferroni correction due to multiple tests. Such an index is preferred to Pearson's r when the variables are (or are likely to be) skewed and/or non-normally distributed. It was expected at least a moderate and positive correlation among BFNE-S and SIAS, SPS, DASS21 Anxiety scores. Similarly, a negative moderate (at least) correlation between BFNE-S and RSES scores was expected. Finally, positive and statistically significant correlations were expected between BFNE-S and the remaining scales. All analyses were performed using the *R* statistical environment (R Core Team, 2020), by using: the *semPower* package (Moshagen, 2021) for sample size estimation, the *lavaan* package (Rosseel, 2012) for CFAs, the psych package (Revelle, 2024) for reliability analyses, and Hmisc (Harrell, 2024) and corrplot (Wei and Simko, 2021) for the other analyses.

## Results

The a priori power analysis suggested that a minimum of 465 respondents were necessary. After the spread on social media, an initial sample consisted of 771 participants. After removing participants with more than 30% missing data, those under 18 years of age, and those with clinical diagnoses, the final sample used for analyses included 652 participants (age: *M* = 27.01, SD = 10.39, range = 18–66 years). The sample was 70.71% female and 29.29% male.

### Confirmatory factor analysis and measurement invariance

The first overall model obtained inadequate fit indices (χ^2^ = 174.197; CFI = 0.992; RMSEA = 0.109). The examination of the modification indices suggested that there is a correlation between items 5 (i.e., “When I am talking to someone, I worry about what they may be thinking about me”) and 6 (i.e., “I am usually worried about what kind of impression I make”) could be the cause of the misfit (MI = 25.35). After including this correlation among items' residuals of the elements in the model structure, the fit improved (χ^2^ =116.939; CFI = 0.995; RMSEA = 0.089). However, further examination of the modification indices suggested that the correlation between items 3 (that is, “I am afraid that others will not approve of me”) and 4 (that is, “I am afraid that other people will find fault with me”) should also be considered (MI = 15.51). Similarly to the previous case, the fit improved (χ^2^ = 81.587; CFI = 0.997; RMSEA = 0.074). Finally, a closer examination of the modification indices suggested that the correlation between items 2 (that is, “I am frequently afraid that other people notice my shortcomings”) and 3 should also be considered (MI = 10.48). After this final inclusion, the CFA model corroborated the unidimensional structure of the BFNE-S, with adequate fit indices (CFI = 0.998, RMSEA = 0.058; standardized factor loadings: Item 1 = 0.82; Item 2 = 0.82; Item 3 = 0.83; Item 4 = 0.88; Item 5 = 0.87; Item 6 = 0.89; Item 7 = 0.85; Item 8 = 0.86). The same was applied after testing all the other models with the proposed structure. It is important to note that the last two correlations also emerged for the single female model (MI = 20.59; 10.40 for Items 3–4 and 2–3, respectively), but not for the single male models. Table 1 reports the results of the final CFA models.

**Table 1:** Fit indices for the BFNE-S models.

**Model**	**χ^2^**	**CFI**	**RMSEA**	**Δ CFI**	**Δ RMSEA**
Overall model	54	0.998	0.058	0.000	0.000
Female model	40	0.998	0.055	0.000	0.000
Male model	46	0.991	0.094	0.000	0.000
Configural model	68	0.995	0.075	0.000	0.000
Metric model	51	0.997	0.061	0.002	0.014
Scalar model	77	0.996	0.052	0.001	0.009

The χ^2^ refers to the scaled one. CFI stands for Comparative Fit Index. RMSEA stands for Root Mean Square Error of Approximation.

### Reliability

BFNE-S showed excellent internal consistency indices, with a Cronbach alpha of 0.96 and McDonald's omega of 0.97; as well as excellent test-retest reliability (ρ = 0.88). The other scales showed similar results, with good Cronbach's alpha and McDonald's omega values for SIAS (α = 0.94, ω = 0.95), SPS (α = 0.95, ω = 0.96), SCOFF (α = 0.95, ω = 0.96), and DASS-21 (total: α = 0.96, ω = 0.97; depression: α = 0.94, ω = 0.96; anxiety: α = 0.90, ω = 0.93; stress: α = 0.91, ω = 0.94).

### Correlations

Statistically significant correlations were observed between the BFNE-S and other scales. BFNE-S was positively correlated with SIAS (*r* = 0.67), SPS (*r* = 0.67), and the depression subscale (*r* = 0.48). The correlations with the anxiety and stress subscales were moderate (*r* = 0.37 and *r* = 0.46, respectively), while the correlation with SCOFF was weaker (*r* = 0.21). BFNE-S was inversely correlated with RSES scores (*r* = −0.53).

## Discussion

The current study sought to provide information about the Italian version of the Brief Fear of Negative Evaluation Scale- Straightforward Items (BFNE-S), specifically to test its factor structure and evaluate its psychometric properties within an Italian, nonclinical sample.

Consistent with previous studies, the current results support the BFNE-S as having a unidimensional factor structure, with excellent fit indices found in the CFA (CFI = 1.000, RMSEA = 0.018) that support structural validity with an Italian sample and among other diverse cultural samples (Pitarch, 2010; Perczel-Forintos and Kresznerits, 2017; Gur Kabul et al., 2023; Wei et al., 2015). The absence of measurement invariance between sexes supports the BFNE-S as robust for males and females, suggesting against the need for sex-specific adaptations.

The factor loadings for the current Italian sample were consistently high, ranging from 0.82 to 0.89, supporting each item as contributing to the latent FNE construct. Pitarch (2010) had reported lower loadings from a Spanish sample (0.57 to 0.75), with item 8 (“I often worry that I will say or do wrong things”) having the lowest loading at 0.57 and Item 7 (“Sometimes I think I am too concerned with what other people think of me”) having the highest loading at 0.75. The results of a sample of Turkish patients with systemic sclerosis by Gur Kabul et al. (2023) also produced lower factor loadings ranging between 0.61 and 0.90 (Item 1 being the lowest at 0.61, whereas Item 4 was the highest at 0.90), and a Chinese adolescent sample (Wei et al., 2015) displayed even lower loadings, from 0.51 (Item 8) to 0.72 (Item 6). The discrepancies in factor loadings may reflect cultural, demographic, or clinical differences that influence perceptions of negative evaluation. In the clinical context of systemic sclerosis, the stigma associated with the condition can further heighten the contributions of specific items (Gur Kabul et al., 2023). Similarly, adolescents navigate a particularly sensitive stage of development, marked by physical, psychological, and social changes. The impact and frequency of peer interactions among adolescents may create particular vulnerabilities for FNE and social anxiety, which may warrant additional attention and specific interventions (Hebert et al., 2013).

Linguistic nuances in the translation of the elements could also affect comprehension and loadings; for example, the phrases “When I am talking to someone, I worry about what they may be thinking about me” (Item 5) and “I am usually worried about what kind of impression I make” (Item 6) are very similar in Chinese, and the items are presented in immediate sequence, which may produce unwarranted covariance and influence the response of the participants (Wei et al., 2015). In the present study, the same correlation was observed. These items are quite similar, both grammatically and semantically, and this could increase the chance of a similar (or equal) response value on the Likert scale. Similarly, the correlation among items 3 (i.e., “I am afraid that others will not approve of me”) and 4 (i.e., “I am afraid that other people will find fault with me”), and 2 (i.e., “I am frequently afraid that other people noticing my shortcomings”) and 3 are worthy of attention. In fact, it could be argued that items 2 and 4 may be perceived as prerequisites of item 3. In other words, it is possible that if people perceive that shortcomings or faults will be found by others, this may lead to a lack of approval from them. Finally, the fact that such correlations were found for the female subsample was interesting, but not in the male one. Future studies could conduct a more detailed item analysis to provide results to this speculation. In any case, the subtle diversities underscore the need for continuous cross-cultural validation of all measures, including the BFNE-S, to support robust use in different social, cultural, and semantic contexts.

The BFNE-S evidenced excellent internal consistency (Cronbach's α = 0.96, McDonald's ω = 0.97), test-retest reliability (ρ = 0.88), and the high correlations with measures of social anxiety (i.e., SIAS and SPS), supporting concurrent validity. Furthermore, these strong associations with SPS and SIAS support the hypothesis that fear of negative evaluation is a central characteristic of social anxiety, corroborating models that position FNE as a transdiagnostic vulnerability factor for interpersonal anxiety (Weeks and Howell, 2012). The high concurrent validity corresponds with previous research that illustrates a positive relation between the BFNE-S and other instruments evaluating social anxiety in American, Chinese, and Spanish samples (Weeks et al., 2005; Wei et al., 2015; Pitarch, 2010). Furthermore, the moderate correlations with the DASS-21 subscales align with the established literature suggesting that, while FNE is related to broader affective states, the construct remains distinct from general distress, possibly due to chronic hypervigilance and self-focused attention associated with evaluative fears (Watson and Friend, 1969; Weeks et al., 2008a). The distinction supports the use of and focus on, the BFNE-S to understand how FNE influences social interactions specifically (Leary and Kowalski, 1995; Utschig et al., 2010; Mancuso et al., 2022; Preston et al., 2023).

The moderate inverse correlation between BFNE-S scores and self-esteem (RSES) is consistent with previous clinical and research observations. We cannot infer causality from a correlation using cross-sectional data, but an increase in FNE can reasonably be expected to correspond with lower self-esteem, which can facilitate further negative thoughts and anxiety (Perczel-Forintos and Kresznerits, 2017). In fact, as expected, the negative correlation between FNE and self-esteem aligns with cognitive-behavioral models of social anxiety, which posit that negative self-evaluations contribute to the anticipation of social failure and fear of being judged (Wells et al., 1995). Individuals with lower self-esteem can perceive themselves as less socially competent or likable, increasing their vulnerability to fears of negative evaluation.

The link with maladaptive eating behaviors, as measured by the SCOFF, further suggests that FNE may contribute to maladaptive coping mechanisms in domains where body image and perceived social scrutiny are particularly salient (Levinson and Rodebaugh, 2012, 2016). This broadens the clinical relevance of BFNE-S beyond anxiety disorders, highlighting its utility in identifying people at risk for a variety of psychological difficulties driven by evaluative concerns.

Given the recent increase in SAD prevalence (Kindred and Bates, 2023), BFNE-S can be an important tool to identify FNE among individuals facing increased social anxiety exacerbated by the pandemic (Kindred and Bates, 2023) and may enhance earlier interventions. Providing a psychometrically robust Italian version of the BFNE-S helps address a gap in the assessment tools available to Italian clinicians and researchers, facilitating more nuanced and culturally sensitive approaches to evaluating and treating SAD. The robust Italian version of the BFNE-S also provides a clear pathway for future additional research on FNE.

## Limitations

The present study supports the use of the limitations of the BFNE-S in Italy, and the study provide important direction for future research. First, participants were recruited through social media, which may skew the sample toward a younger, more technologically savvy demographic, which would not fully reflect the Italian population. Second, cross-sectional data collection precludes discussions of causality and limits assessments of criterion validity. The current results support future investments in longitudinal study designs. Third, despite the absence of measurement invariance between sexes, the sample was imbalanced with respect to sex (that is, 71% female, 29% male). This imbalance could also explain some differences in CFA models (e.g., the fact that some items correlate only for the female subsample, creating potential misfit issues). In terms of overall fit, it could be argued that values of CFI extremely close to 1 could increase the risk of untestable models (since they are saturated or over-saturated). Nonetheless, it is well known that with large sample (>100) size as in the present study (*n* > 600), CFI is less sensitive than RMSEA (Taasoobshirazi and Wang, 2016). Future researchers could benefit from broader sampling methods to further support representativeness and generalizability. Fourth, only participants without psychological diagnoses were included for the present study, since our primary objective was to evaluate the applicability within a non-clinical sample; however, the clinical diagnostic status was determined by self-report, which means that participants without diagnoses who nevertheless experienced psychological challenges may have been included.

## Conclusions

The BFNE-S appears psychometrically robust for use with Italian samples. The current results have supported cross-cultural applicability, providing clinicians and researchers in Italy with a reliable tool for assessing the components of social anxiety related to FNE. BFNE-S appears to be a valuable tool for both therapeutic and research applications, contributing to evaluation and treatment efforts, and to experimental efforts toward understanding FNE as a construct in diverse populations.

## Data Availability

Data are available on reasonable requests to the corresponding author.

## Appendix

**Table A1:** The scale inter-correlations.

**Scale 1**	**Scale 2**	**ρ**	***P* adjusted**
BFNE-S	RSES	−0.534	< 0.001
BFNE-S	D21_Dep	0.475	< 0.001
BFNE-S	D21_Anx	0.373	< 0.001
BFNE-S	D21_Str	0.460	< 0.001
BFNE-S	D21_Tot	0.488	< 0.001
BFNE-S	SIAS	0.671	< 0.001
BFNE-S	SPS	0.673	< 0.001
BFNE-S	SCOFF	0.213	< 0.001

BFNE-S, Brief Fear of Negative Evaluation Straightforward; RSES, Rosenberg Self-Esteem Scale; D21_Dep, DASS-21 Depression Scale; D21_Anx, DASS-21 Anxiety Scale; D21_Str, DASS-21 Stress Scale; D21_Tot, Depression Anxiety and Stress Scale Total Score; SIAS, Social Interaction Anxiety Scale; SPS, Social Phobia Scale; SCOFF, Sick Control One Fat Food Questionnaire.
